# Impact of the Japanese clinical practice guidelines for management of sepsis and septic shock (J-SSCG) 2020 on real-world adherence and interhospital variation: a nationwide inpatient database study

**DOI:** 10.1186/s13054-025-05482-9

**Published:** 2025-06-03

**Authors:** Hiroyuki Ohbe, Kazuma Yamakawa, Daisuke Kudo, Shotaro Aso, Hiroki Matsui, Kiyohide Fushimi, Hideo Yasunaga, Tomoaki Yatabe, Moritoki Egi, Hiroshi Ogura, Osamu Nishida, Shigeki Kushimoto

**Affiliations:** 1 Department of Emergency and Critical Care Medicine, Tohoku University Hospital, 1-1 Seiryo-machi, Aoba-ku, Sendai, 980-8574 Japan; 2 Department of Clinical Epidemiology and Health Economics, School of Public Health, The University of Tokyo, 7-3-1 Hongo, Bunkyo-ku, Tokyo, 113-0033 Japan; 3 Department of Emergency and Critical Care Medicine, Osaka Medical and Pharmaceutical University, 2-7 Daigaku-machi, Takatsuki, 569-8686 Osaka Japan; 4 Division of Emergency and Critical Care Medicine, Tohoku University Graduate School of Medicine, 2-1 Seiryo-machi, Aoba-ku, Sendai, 980-8575 Miyagi Japan; 5 Department of Health Services Research, Graduate School of Medicine, The University of Tokyo, 7-3-1, Hongo, Bunkyo-ku, Tokyo, 113-0033 Japan; 6 Department of Health Policy and Informatics, Institute of Science Tokyo Graduate School, 2-12-1 Ookayama, Meguro-ku, Tokyo, 152-8550 Japan; 7 Emergency Department, Nishichita General Hospital, 3-1-1 Nakanoike, Tokai-shi, 477-8522 Aichi Japan; 8 Department of Anesthesia and Intensive Care, Kyoto University Hospital, 54 Kawahara-cho, Shogoin, Sakyo-ku, Kyoto, 606-8507 Japan; 9 Department of Clinical Laboratory, Osaka General Medical Center, 3-1-56 Bandai-Higashi, Sumiyoshi-ku, Osaka-shi, 558-8558 Osaka Japan; 10 Department of Anesthesiology and Critical Care Medicine, Fujita Health University, 1-98 Dengakugakubo, Kutsukake, Toyoake, 470-1192 Aichi Japan; 11 Executive Advisor for Emergency Medicine and Intensive Care, Kawasaki Saiwai Hospital, 31-27 Omiya-cho, Saiwai-ku, Kawasaki, 212-0014 Kanagawa Japan

**Keywords:** Sepsis, Clinical practice guidelines, Adherence, Interrupted time series, Hospital variation

## Abstract

**Background:**

The Japanese Clinical Practice Guidelines for Management of Sepsis and Septic Shock (J-SSCG) 2020 aimed to standardize sepsis care in Japan. However, the extent of their impact on clinical practice remains uncertain.

**Methods:**

We conducted a nationwide retrospective cohort study using the Japanese Diagnosis Procedure Combination database between April 2018 and December 2021. Of the 118 clinical questions (CQs) in the J-SSCG 2020, we identified 26 recommendations to which adherence could be evaluated using patient-level data. We evaluated adherence trends before and after the guideline’s publication using interrupted time series analysis and quantified hospital-level variation using intraclass correlation coefficients.

**Results:**

A total of 213,099 patients with sepsis from 791 hospitals were included. Adherence rates varied widely across CQs (range: 0.5–98.7%). Recommendations “against” interventions generally showed high adherence, whereas those “for” interventions exhibited lower and more variable adherence. After guideline publication, adherence increased by < 3% points for most CQs. Interrupted time series analysis demonstrated no abrupt or substantial changes, and statistically significant trends were modest (< 2% annually). Among the 26 CQs, 14 were consistent with J-SSCG 2016 and 12 were newly introduced in 2020; both groups showed similarly limited changes in adherence. Adjusted intraclass correlation coefficients exceeded 10% for 22 CQs, indicating persistent between-hospital variation, which remained unchanged after the guideline’s release.

**Conclusions:**

This nationwide study identified persistent evidence–practice gaps, minimal improvements in adherence after J-SSCG 2020, and substantial interhospital variation that remained unaltered. These findings underscore the challenges of implementing guidelines in practice and highlight the need to better understand contextual barriers to standardized sepsis care in Japan.

**Supplementary Information:**

The online version contains supplementary material available at 10.1186/s13054-025-05482-9.

## Background

Sepsis remains a leading cause of morbidity and mortality worldwide [1]. In response to the global burden of sepsis, clinical practice guidelines—such as the Surviving Sepsis Campaign: International Guidelines for Management of Sepsis and Septic Shock (SSCG)—have been developed to promote standardized, evidence-based care [2]. In Japan, the Japanese Society of Intensive Care Medicine and the Japanese Association for Acute Medicine published the Japanese Clinical Practice Guidelines for Management of Sepsis and Septic Shock (J-SSCG) in 2012, with revisions in 2016, 2020, and 2024 [3–6].

Despite the availability of high-quality guidelines, their impact on clinical practice remains uncertain. A systematic review of 235 studies found that guideline dissemination often results in modest changes in physician behavior, typically with improvements of < 10% [7, 8]. Studies across various disease domains—including cardiovascular disease, diabetes, and atrial fibrillation—have revealed persistent gaps between evidence-based recommendations and clinical practice [9–11]. This “evidence–practice gap” poses a major barrier to improving care quality and patient outcomes [8].

Understanding the magnitude and underlying causes of the evidence–practice gap is critical for successful guideline implementation. However, few studies in Japan have assessed the extent to which clinical practice aligns with J-SSCG recommendations, especially those introduced in 2020. A multicenter study conducted between June 2010 and December 2011 across 39 hospitals (*n* = 1,104) evaluated the impact of SSCG 2008 and reported compliance rates of 11.2% for the complete sepsis resuscitation bundle and 39.7% for the complete sepsis management bundle [12]. Another multicenter study, involving 1,184 patients in 59 intensive care units (ICUs) between January 2016 and March 2017, examined adherence to SSCG 2012 and J-SSCG 2012 [13]. This study found relatively high compliance with the 3-hour bundle at 64.3%, including 96.9% for serum lactate measurement and 76.3% for fluid resuscitation; however, compliance with the full 6-hour bundle was considerably lower at 3.5%, despite high adherence to individual components such as vasopressor use (88.6%) and repeat lactate measurement (90.0%) [13]. The same group reported that 73.9% of patients received antibiotics within 3 h of sepsis recognition [14].

Although these studies offer insights into the evidence–practice gap in sepsis care, they predate J-SSCG 2020 and do not reflect its updated recommendations. Moreover, the study hospitals were primarily institutions with a strong focus on sepsis care, potentially limiting generalizability to the broader hospital population. Study durations were also relatively short (≤ 19 months), limiting their ability to assess practice changes before and after the introduction of SSCG or J-SSCG. Additionally, hospital-level variation in adherence—a key factor in understanding implementation challenges—has not been quantitatively assessed in previous studies.

Therefore, this study aimed to evaluate adherence with key J-SSCG 2020 recommendations, assess the impact of the guideline’s publication using interrupted time series (ITS) analysis from 2018 to 2021, and examine hospital-level variation in adherence.

## Methods

### Study design and data source

This nationwide retrospective cohort study utilized data from the Diagnosis Procedure Combination (DPC) database and the Hospital Bed Function Report [15] in Japan. The DPC database comprises discharge abstracts and administrative claims from voluntarily participating hospitals, representing over 1,500 acute-care hospitals and encompassing approximately 50% of all acute-care hospital beds, 75% of all ICU beds, and 70% of all HDU beds in Japan [16]. It includes patient-level data for all hospitalizations, such as demographic characteristics, diagnoses coded using the International Classification of Diseases, Tenth Revision (ICD-10), daily procedures recorded with Japanese procedure codes, daily drug administrations, and admission and discharge status. A previous validation study demonstrated high specificity and moderate sensitivity for recorded diagnoses, as well as high specificity and sensitivity for recorded procedures [17]. Interventions performed in the emergency department were included as day 1 (the day of admission) in patient admitted through the emergency department.

Since April 1, 2018, healthcare providers in Japan have been required to enter Sequential Organ Failure Assessment (SOFA) scores into the DPC database on the first day of sepsis treatment for all patients aged ≥ 15 years, regardless of care unit [18]. The worst total SOFA score on the first day of sepsis treatment is recorded in the database, with scores ranging from 0 (normal) to 4 (high degree of dysfunction/failure) for each of six organ systems.

We also used facility-level data from the Hospital Bed Function Report (2018–2021), published annually by the Ministry of Health, Labour, and Welfare in Japan, which includes statistics as of July 1 each year [15]. These data were linked to the DPC database using a hospital-specific identifier. The report provides details such as ward type (e.g., general ward, ICU, or high-dependency unit [HDU], number of beds per ward, and hospital classification (e.g., teaching hospital, academic hospital, or tertiary emergency hospital).

This study was approved by the Institutional Review Board of The University of Tokyo [approval number: 3501-(5) (May 19 th, 2021)]. Because all data were de-identified, the requirement for informed consent was waived. All procedures were conducted in accordance with the Declaration of Helsinki.

## Study population

We identified all patients aged ≥ 15 years diagnosed with sepsis and had SOFA scores recorded between April 1, 2018, and December 31, 2021. We excluded patients who (i) were aged < 15 years, (ii) had a total SOFA score < 2 on the first day of sepsis treatment based on the Sepsis-3 definition [19], or (iii) were treated in hospitals that did not record at least five patients with sepsis annually throughout the 4-year study period (fiscal year 2021 was 9 months). In this study, we imputed missing SOFA score variables as 0, assuming no organ dysfunction, in accordance with the approach used in the Sepsis-3 guidelines [19]. Septic shock was defined as the administration of any vasopressor (noradrenaline, adrenaline, dopamine, vasopressin, or phenylephrine) on the first day of sepsis treatment, given the lack of mean arterial pressure and lactate data in the DPC database. All patients were followed up until in-hospital death or discharge.

## Japanese clinical practice guidelines management for Sepsis and septic shock

The original Japanese version of the J-SSCG 2020 was first published online ahead of print on the official websites of the Japanese Society of Intensive Care Medicine and the Japanese Association for Acute Medicine on September 30, 2020. Subsequently, it was published in the official society journal on February 25, 2021 [5]. Among the 118 clinical questions (CQs) in J-SSCG 2020 [5], 26 (22.0%) were deemed assessable using the DPC database following discussion between clinical experts (HO and KY). These included: 1 in CQ2 (Diagnosis of infection), 1 in CQ4 (Antimicrobial therapy), 1 in CQ5 (Intravenous immunoglobulin therapy), 8 in CQ6 (Initial resuscitation/inotropes), 3 in CQ7 (Corticosteroid therapy), 1 in CQ10 (Management of pain, agitation, and delirium), 4 in CQ11 (Acute kidney injury/blood purification), 2 in CQ12 (Nutrition support therapy), 2 in CQ15 (Diagnosis and treatment of disseminated intravascular coagulation in patients with sepsis), 1 in CQ17 (ICU-acquired weakness and early rehabilitation), 1 in CQ21 (Sepsis treatment system), and 1 in CQ22 (Stress ulcer prophylaxis). Adherence was defined according to the direction of each J-SSCG 2020 recommendation. For recommendations advising against interventions, non-use was considered adherence. The adherence for each CQ was defined using the numerator and denominator described in Supplementary material 1: Table S1. Recommendation strength was classified as strong (GRADE 1 or good practice statement) or weak (GRADE 2 or expert consensus) according to the published GRADE guidance [20].

## Other variables and outcomes

Patient characteristics included age, sex, hospital-acquired sepsis, surgery with general anesthesia, infection type, and total SOFA score on the day of sepsis treatment. ICD-10 codes for infection types are provided in Supplementary material 1: Table 2. Clinical outcomes included in-hospital mortality, length of stay, and total hospitalization costs.

## Statistical analyses

We first conducted descriptive analyses comparing patient characteristics, outcomes, and proportions of patients receiving care consistent with each assessable CQ before and after publication of the J-SSCG 2020. The pre- and post-guideline periods were defined as April 1, 2018, to September 30, 2020 (30 months), and October 1, 2020, to December 31, 2021 (15 months), respectively. Continuous variables were reported as means and standard deviations or medians and interquartile ranges; categorical variables were reported as numbers and percentages, as appropriate. Due to the large sample size, standardized mean differences (SMDs) were used for group comparisons, with an absolute SMD ≤ 10% considered indicative of negligible imbalance [21].

To evaluate the temporal impact of J-SSCG 2020 publication, we conducted ITS analyses using segmented linear regression [22]. The outcome variable was the monthly proportion of patients receiving guideline-concordant care across all CQs. The models estimated the pre-guideline trend, level change, and slope change following guideline publication, with 95% confidence intervals. The unit of analysis was month, yielding 45 time points (30 pre-guideline and 15 post-guideline). Although several recommendations in the J-SSCG 2020 were retained from the 2016 version of the SSCG and J-SSCG, our ITS design enabled us to assess changes in adherence that were specifically associated with the 2020 guideline publication, while explicitly accounting for underlying pre-guideline trends. As a sensitivity analysis, we redefined the post-guideline period from March 1, 2021 to December 31, 2021, to account for a potential uptake period following the dissemination of the J-SSCG 2020.

In addition, we assessed the overall impact of the J-SSCG 2020 publication on clinical outcomes using ITS analysis. First, monthly adjusted outcomes of in-hospital mortality, length of stay, and hospitalization costs were estimated using multilevel logistic or linear regression models with a random intercept for hospitals, adjusting for age, sex, hospital-acquired sepsis, surgery, infection type, and SOFA score [23]. These adjusted outcomes were then analyzed based on the same ITS framework.

To assess hospital-level variation in J-SSCG 2020 adherence, we calculated intraclass correlation coefficients (ICCs) using multilevel logistic regression with a random intercept for hospitals [23]. ICCs were estimated separately for the pre- and post-guideline periods and were reported as both unadjusted and adjusted for patient-level covariates (age, sex, hospital-acquired sepsis, surgery with general anesthesia, infection type, and SOFA score on the day of treatment). An ICC of 0% indicates no hospital-level variation, whereas values closer to 100% suggest care was primarily influenced by hospital characteristics [23]. ICCs ≥ 5–10% are generally considered to represent meaningful between-hospital variation [23].

All statistical analyses were conducted using Stata/MP 18.0 (StataCorp, College Station, TX, USA). All reported *P*-values were two-sided, and a *P* value of < 0.05 was considered statistically significant.

## Results

### Study population patient characteristics, and outcomes

**Fig. 1:**
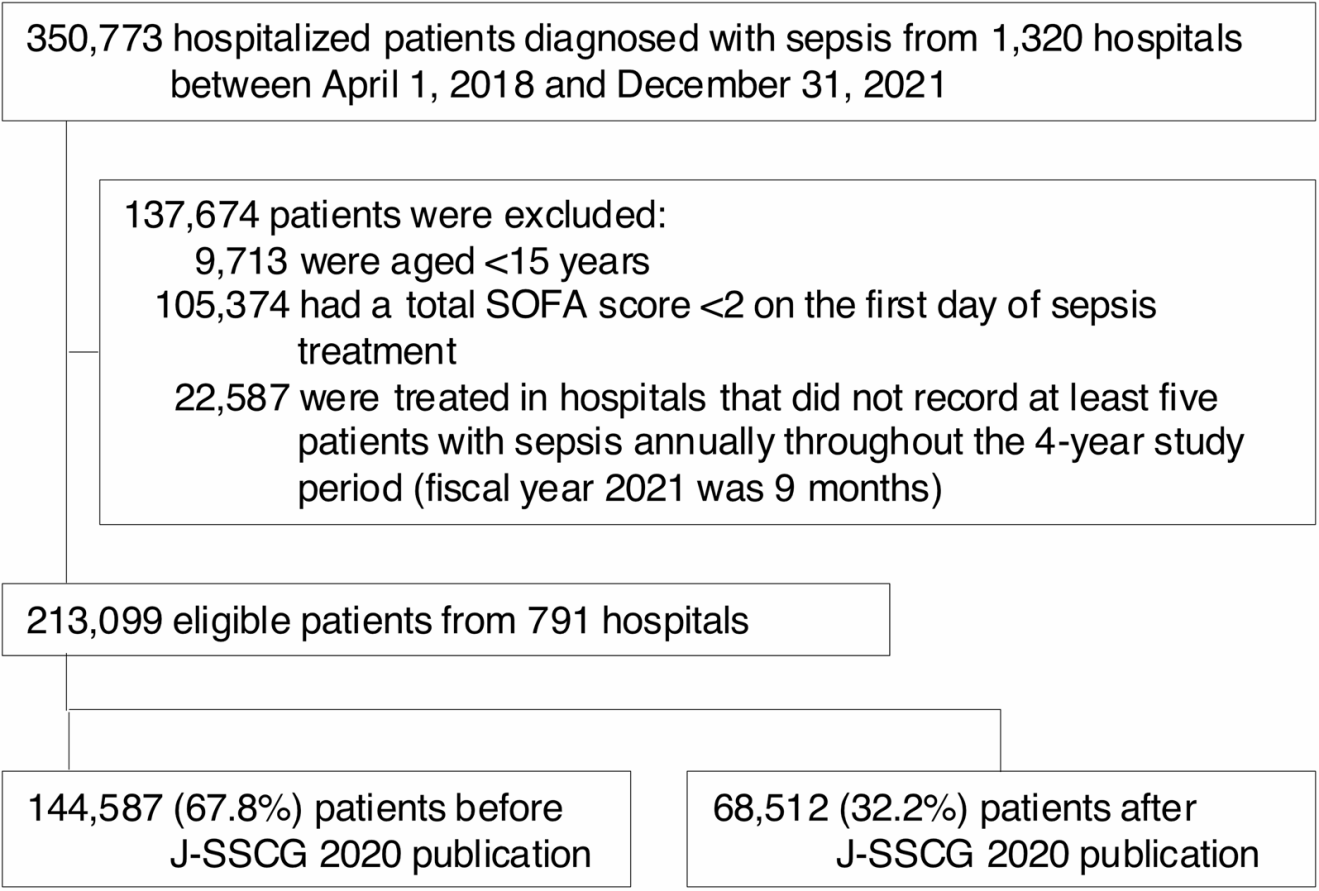
Patient flowchart SOFA, Sequential Organ Failure Assessment; J-SSCG, Japanese Clinical Practice Guidelines for Management of Sepsis and Septic Shock

A total of 213,099 patients diagnosed with sepsis were included in the analysis, of whom 144,587 (67.8%) were hospitalized during the pre-guideline period and 68,512 (32.2%) during the post-guideline period (Fig. 1). Throughout the study period (2018–2021), 791 hospitals consistently contributed data each year (Supplementary material 1: Table S3). Most were teaching hospitals (84%), with a smaller proportion being academic hospitals (9%) and tertiary emergency centers (27%). Hospital characteristics remained stable throughout the study period. The overall mean age was 76.3 years, and 56.1% of patients were male (Table 1). Hospital-acquired sepsis occurred in 31.6% of cases, and 11.0% of patients underwent surgery under general anesthesia. The in-hospital mortality rate was 29.7% and median length of hospital stay was 22.0 days. All standardized mean differences for hospital and patient characteristics and outcomes were below 10%, indicating adequate balance between the pre- and post-guideline groups.

**Table 1:** Patient characteristics before and after J-SSCG 2020 publication

Characteristics	Overall *N* = 213,099	Before J-SSCG2020 *N* = 144,587	After J-SSCG2020 *N* = 68,512	SMD
Age, years	76.3 (13.3)	76.1 (13.4)	76.8 (13.2)	5.7
Male	119,447 (56.1)	81,145 (56.1)	38,302 (55.9)	−0.4
Hospital-acquired sepsis	67,314 (31.6)	46,617 (32.2)	20,697 (30.2)	−4.4
Surgery with general anesthesia	23,505 (11.0)	15,912 (11.0)	7,593 (11.1)	0.2
Infection Type				
Lung	44,563 (20.9)	30,998 (21.4)	13,565 (19.8)	−4.1
Abdomen	42,073 (19.7)	28,346 (19.6)	13,727 (20.0)	1.1
Urinary tract	41,541 (19.5)	27,622 (19.1)	13,919 (20.3)	3.0
Central nervous system	1,733 (0.8)	1,231 (0.9)	502 (0.7)	−1.3
Skin and soft tissues	6,155 (2.9)	4,259 (2.9)	1,896 (2.8)	−1.1
Cardiovascular system	5,841 (2.7)	3,896 (2.7)	1,945 (2.8)	0.9
Others	86,889 (40.8)	58,983 (40.8)	27,906 (40.7)	−0.1
Total SOFA score	5.0 (3.0–8.0)	5.0 (3.0–8.0)	5.0 (3.0–8.0)	3.0
Outcomes				
In-hospital mortality	67,317 (31.6)	45,083 (31.2)	22,234 (32.5)	2.7
Length of hospital stay, days	22.0 (12.0–43.0)	23.0 (12.0–45.0)	21.0 (11.0–41.0)	−6.6
Hospitalization costs, million yen	1.3 (0.7–2.6)	1.3 (0.7–2.7)	1.3 (0.7–2.6)	−2.7

Values are presented as mean (standard deviation), median (interquartile range), or number (percentage), as appropriate. Comparisons reflect the pre-guideline period (April 1, 2018 to September 30, 2020) and the post-guideline period (October 1, 2020 to December 31, 2021)

SMD, standardized mean difference; J-SSCG, Japanese Clinical Practice Guidelines for Management of Sepsis and Septic Shock; SOFA, Sequential Organ Failure Assessment

## Adherence to J-SSCG 2020 CQs

Among the 26 assessable CQs, adherence rates varied widely across domains and specific interventions, ranging from 0.5 to 98.7% (Table 2). Regarding directionality, 16 CQs were recommendations “for” the intervention and 10 were “against.” Of the 26 CQs, only two were strong recommendations, whereas the remaining 24 were weak recommendations.

**Table 2:** Adherence to clinical questions before and after publication of the J-SSCG 2020

CQ	Recommendation Direction	Number of eligible patients	Adherence Before J-SSCG2020	Adherence After J-SSCG2020	SMD
CQ2-1: When should a blood culture be taken?	GPS, Strong, For	213,099	116,846/144,587 (81.1)	55,961/68,512 (81.7)	2.2
CQ4-9: Should PCT be used as an indicator for stopping antimicrobial therapy for sepsis?	GRADE 2B, Weak, For	184,822	12,931/126,140 (10.4)	6,320/58,682 (10.8)	1.7
CQ5-1: Should intravenous immunoglobulin be administered to adult patients with sepsis?	GRADE 2B, Weak, Against	213,099	127,379/144,587 (89.0)	62,178/68,512 (90.8)	8.6
CQ6-1: Should echocardiography be conducted in patients with sepsis?	GRADE 2D, Weak, For	82,487	13,379/54,143 (24.7)	6,995/28,344 (24.7)	−0.1
CQ6-4: Should lactate levels be used as an indicator for initial resuscitation in adult patients with sepsis?	GRADE 2 C, Weak, For	82,487	44,248/54,143 (81.9)	23,339/28,344 (82.3)	1.6
CQ6-7: Should albumin solution be used for initial resuscitation in adult patients with sepsis?	GRADE 2 C, Weak, Against	82,487	41,652/54,143 (77.2)	22,053/28,344 (77.8)	2.1
CQ6-8: Should artificial colloids be used for initial resuscitation in adult patients with sepsis?	GRADE 2D, Weak, Against	82,487	49,715/54,143 (92.2)	26,361/28,344 (93.0)	4.5
CQ6-9-1: Should noradrenaline, dopamine, or phenylephrine be used as a first-line vasopressor in adult patients with sepsis? noradrenaline vs. dopamine	GRADE 2D, Weak, For	79,208	46,712/51,886 (91.2)	25,543/27,322 (93.5)	12.6
CQ6-9-2: Should noradrenaline, dopamine, or phenylephrine be used as a first-line vasopressor in adult patients with sepsis? noradrenaline vs. phenylephrine	GRADE 2D, Weak, For	74,864	46,712/48,530 (96.5)	25,543/26,334 (97.0)	4.1
CQ6-10-1: Should adrenaline be used as a second-line vasopressor in adult patients with sepsis?	GRADE 2D, Weak, Against	15,845	6,844/9,964 (70.5)	4,322/5,881 (73.5)	10.6
CQ6-10-2: Should vasopressin be used as a second-line vasopressor in adult patients with sepsis?	GRADE 2D, Weak, For	15,845	7,875/9,964 (81.1)	4,971/5,881 (84.5)	14.3
CQ7-1: Should low-dose corticosteroids (hydrocortisone) be administered to adult patients with septic shock who do not respond to initial fluid resuscitation and vasopressors?	GRADE 2D, Weak, For	15,845	5,246/9,964 (54.0)	3,310/5,881 (56.3)	7.3
CQ7-2: Should hydrocortisone and fludrocortisone be administered to patients with septic shock who do not respond to initial fluid resuscitation and vasopressors?	GRADE 2 C, Weak, For	8,556	18/5,246 (0.5)	22/3,310 (0.7)	4.5
CQ7-3: Should corticosteroids (hydrocortisone) be administered to patients with sepsis without shock?	GRADE 2D, Weak, Against	130,612	87,360/90,444 (96.6)	38,839/40,168 (96.7)	0.6
CQ10-2: Should propofol or dexmedetomidine be prioritized over benzodiazepines as sedatives for adult patients with sepsis on mechanical ventilation?	GRADE 2D, Weak, For	46,153	19,544/31,444 (62.8)	9,437/14,709 (64.2)	4.2
CQ11-1: Should furosemide be used to prevent or treat septic AKI?	GRADE 2 C, Weak, Against	113,643	66,591/76,133 (87.8)	33,165/37,510 (88.4)	2.9
CQ11-2: Should atrial natriuretic peptide (ANP) be used to prevent or treat septic AKI?	GRADE 2D, Weak, Against	113,643	74,852/76,133 (98.5)	37,031/37,510 (98.7)	3.4
CQ11-3: Should dopamine be used to prevent or treat septic AKI?	GRADE 2 C, Weak, Against	113,643	68,334/76,133 (90.6)	34,625/37,510 (92.3)	8.9
CQ11-7: Should PMX-DHP be used for patients with septic shock?	GRADE 2B, Weak, Against	82,487	51,039/54,143 (94.8)	27,166/28,344 (95.8)	7.3
CQ12-2: Should hemodynamically unstable septic shock patients receive enteral nutrition?	GRADE 2D, Weak, Against	82,487	41,190/54,143 (76.3)	21,755/28,344 (76.8)	1.6
CQ12-3: When should enteral nutrition be initiated in septic patients?	GRADE 2D, Weak, For	213,099	72,311/144,587 (49.3)	32,839/68,512 (47.9)	−4.2
CQ15-3: Should antithrombin replacement therapy be administered in sepsis-associated DIC?	GRADE 2 C, Weak, For	38,138	5,774/26,514 (21.4)	2,391/11,624 (20.6)	−3.0
CQ15-5: Should recombinant thrombomodulin be administered to patients with sepsis-associated DIC?	GRADE 2 C, Weak, For	38,138	16,170/26,514 (59.9)	6,656/11,624 (57.3)	−7.6
CQ17-1: Should early rehabilitation be implemented to prevent PICS?	GRADE 2D, Weak, For	213,099	66,969/144,587 (48.1)	35,512/68,512 (51.8)	11.1
CQ21-3: Where should sepsis which does not respond to initial fluid resuscitation be managed?	GPS, Strong, For	82,487	22,409/54,143 (40.7)	11,155/28,344 (39.4)	−4.1
CQ22-1: Should antiulcer drugs be administered to septic patients to prevent gastrointestinal bleeding?	GRADE 2B, Weak, For	213,099	106,731/144,587 (73.9)	50,687/68,512 (74.0)	0.4

J-SSCG, Japanese Clinical Practice Guidelines for Management of Sepsis and Septic Shock; SMD, standardized mean difference; CQ, Clinical Question; GPS, Good Practice Statement; PCT, Procalcitonin; AKI, Acute Kidney Injury; PMX-DHP, Polymyxin B-immobilized fiber direct hemoperfusion; DIC, Disseminated Intravascular Coagulation; PICS, Post-Intensive Care Syndrome

Adherence to “for” recommendations was generally lower, with only 4 of 16 CQs (25%) achieving adherence rates ≥ 80%. Four high adherence interventions included blood culture (CQ2-1, 81.1–81.7%), noradrenaline as the first-line vasopressor (CQ6–9–1 and CQ6–9–2, 91.2–97.0%), and vasopressin as the second-line vasopressor (CQ6–10–2, 81.1–84.5%). Conversely, several “for” recommendations demonstrated limited adherence, such as procalcitonin (CQ4–9, 10.4–10.8%), echocardiography (CQ6–1, 24.7%), combined hydrocortisone and fludrocortisone use (CQ7–2, 0.5–0.7%), antithrombin for sepsis-associated DIC (CQ15–3, 20.6–21.4%), and ICU admission for septic shock (CQ21–3, 39.4–40.7%).

Adherence to “against” recommendations was generally high, with all 10 “against” CQs (100%) demonstrating adherence rates ≥ 70%, and 8 of 10 (80%) exceeding 80%. High adherence was observed for avoiding artificial colloids for resuscitation (CQ6–8, 92.2–93.0%), dopamine for AKI (CQ11–3, 90.6–92.3%), atrial natriuretic peptide (CQ11–2, 98.5–98.7%), and PMX-DHP (CQ11–7, 94.8–95.8%). Only two CQs were strong recommendations, with high adherence to blood culture collection (CQ, 2–1; 81.1–81.7%) and low adherence to ICU admission for septic shock (CQ, 21–3; 39.4–40.7%).

Among the 26 CQs, four—CQ6–9–1 (noradrenaline as a first-line vasopressor over dopamine), CQ6–10–1 (adrenaline as second-line vasopressor), CQ6–10–2 (vasopressin as second-line vasopressor), and CQ17–1 (early rehabilitation)—had standardized mean differences > 10% between the pre- and post-guideline periods. However, absolute adherence increases in the post-guideline period were modest, approximately 3% points.

### Temporal trends in adherence: ITS analysis

Before evaluating the impact of the J-SSCG 2020 publication on adherence trends, it is important to consider the continuity of the recommendations. Supplementary material 1: Table S4 indicates that 14 CQs were retained from the J-SSCG 2016, while 12 were newly introduced in 2020.

**Fig. 2:**
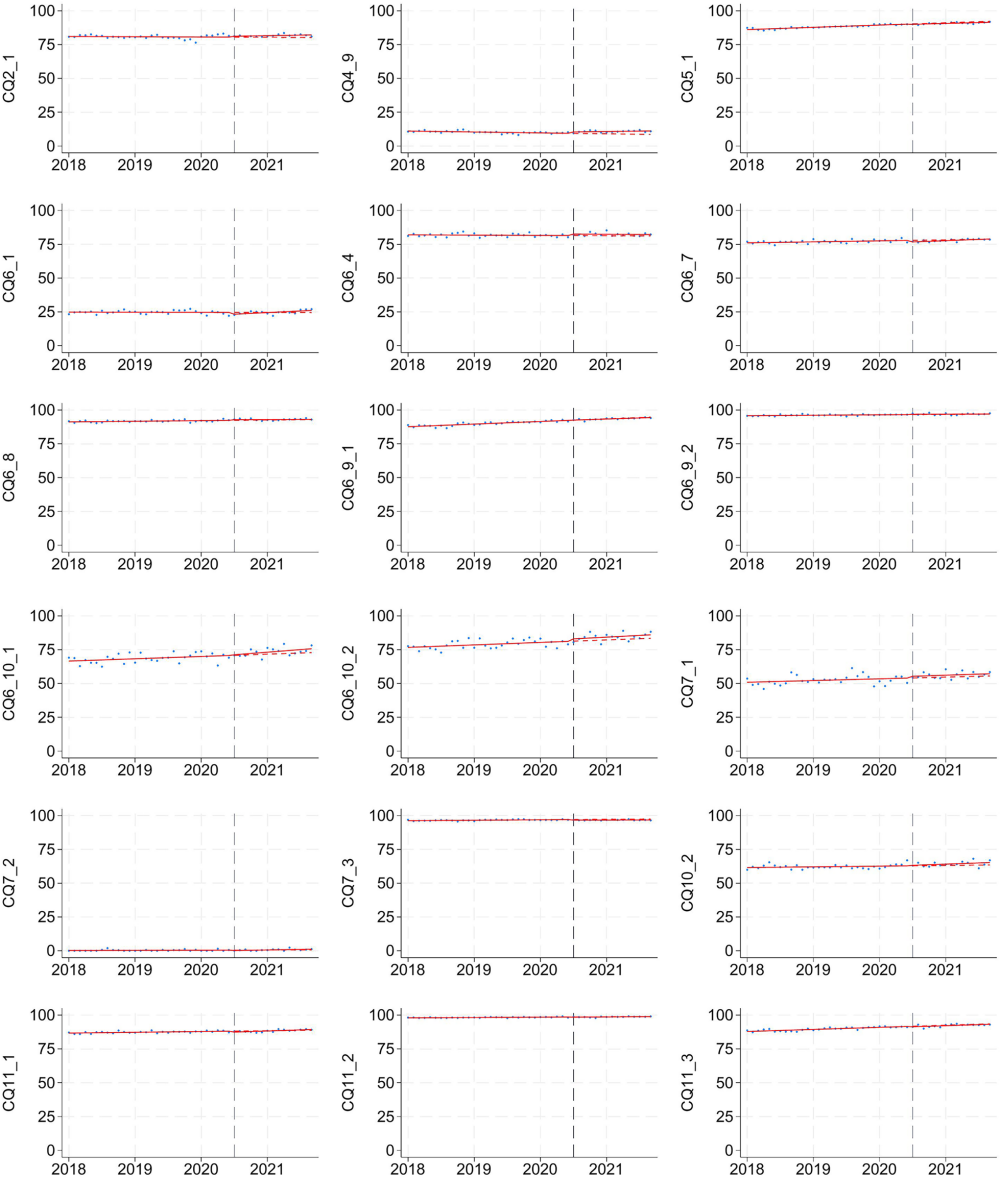

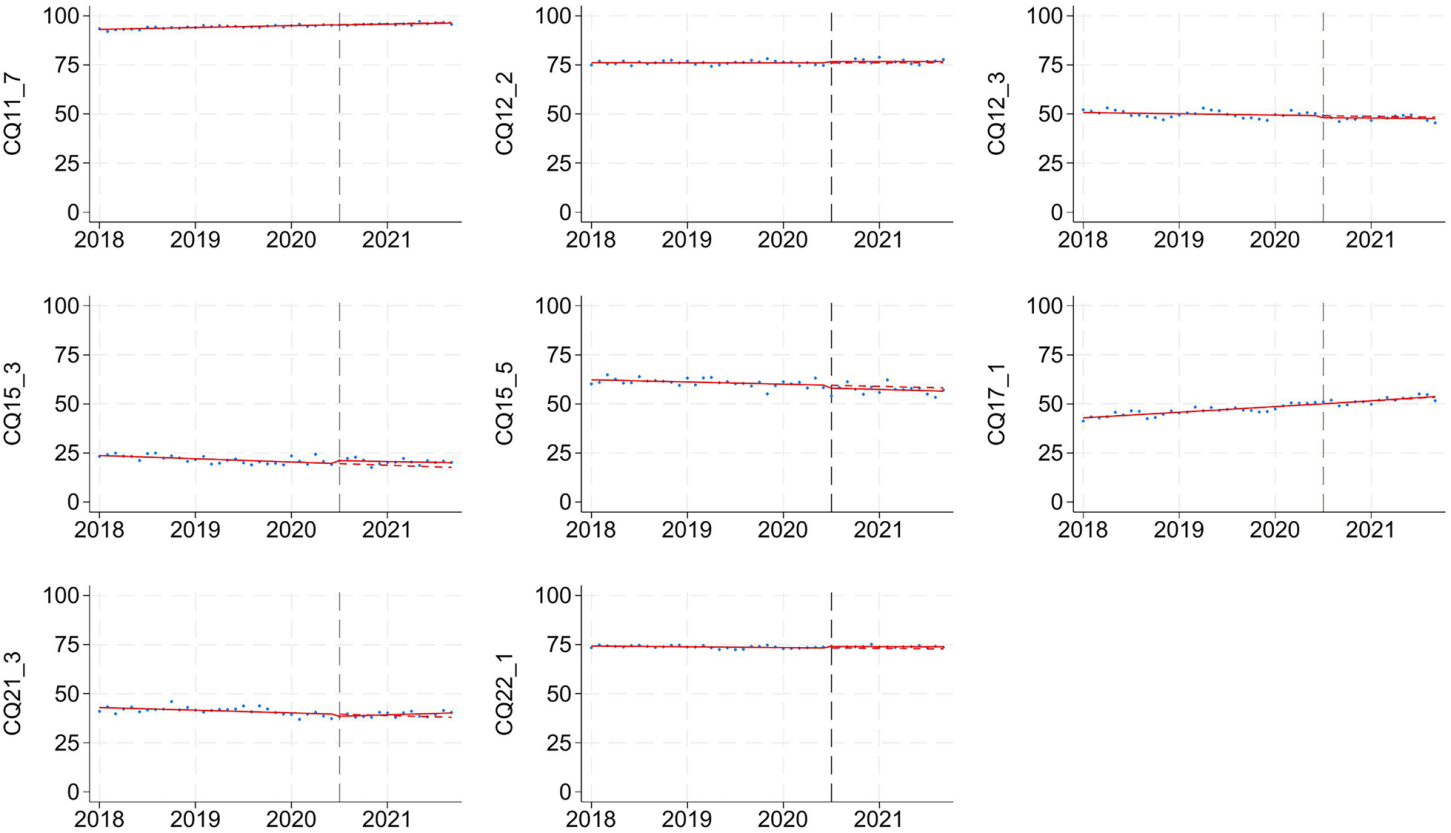
Temporal trends in guideline adherence for clinical questions before and after the publication of the J-SSCG 2020 The vertical dashed line indicates the publication date of the guideline (October 2020). Data points represent monthly adherence rates, and solid red lines represent fitted segmented regression models from interrupted time series analysis. The red dashed line depicts the projected adherence trend after October 2020, assuming the pre-guideline trend had continued unchanged. Each panel corresponds to one CQ and presents adherence trends over the study period (April 2018 to December 2021)

Temporal adherence trends were analyzed using ITS (Table 3). Statistically significant changes in level or slope were observed for a subset of CQs, including CQ4–9 (procalcitonin), CQ6–1 (echocardiography), CQ6–7 (albumin), CQ7–2 (fludrocortisone), CQ11–1 (furosemide), CQ21–3 (ICU admission), and CQ22–1 (antiulcer). However, these changes were small, with level changes ranging from 0.8 to 1.0% and annual slope changes from 0.7 to 2.8%. Among the four CQs showing post-guideline increases with standardized mean differences > 10% (Table 1; CQ6–9–1, CQ6–10–1, CQ6–10–2, and CQ17–1), no statistically significant changes were observed in the ITS analysis, suggesting that these increases likely reflected ongoing trends rather than effects of the guideline publication. Figure 2 illustrates that no CQs showed abrupt or substantial adherence shifts after publication. Using a sensitivity analysis selecting March 1 to December 31, 2021 as the post-guideline period, adherence trends remained similar. The ITS results were consistent, supporting the robustness of our main findings (Supplementary material 1: Table S5).

**Table 3:** Interrupted time series analysis of Temporal trends in adherence to clinical questions before and after J-SSCG 2020 publication

CQ	Pre-trend (95% CI)	*P value*	Level-change (95% CI)	*P value*	Slope change (95% CI)	*P value*
CQ2-1	−0.2 (−1.0, 0.6)	0.611	0.7 (−0.8, 2.1)	0.375	1.0 (−0.2, 2.3)	0.100
CQ4-9	−0.7 (−1.0, −0.4)	0.000	1.0 (0.1, 1.9)	0.025	1.3 (0.3, 2.4)	0.016
CQ5-1	1.7 (1.3, 2.1)	0.000	−0.3 (−0.9, 0.3)	0.324	−0.4 (−1.1, 0.3)	0.264
CQ6-1	−0.1 (−0.8, 0.6)	0.816	−1.3 (−2.8, 0.2)	0.082	2.4 (0.9, 4.0)	0.002
CQ6-4	−0.2 (−0.8, 0.3)	0.445	1.1 (−0.3, 2.4)	0.121	−0.1 (−1.5, 1.3)	0.924
CQ6-7	0.7 (0.2, 1.3)	0.011	−1.2 (−2.2, −0.1)	0.029	1.1 (0.2, 2.1)	0.020
CQ6-8	0.5 (0.1, 0.8)	0.016	0.5 (−0.4, 1.3)	0.271	−0.3 (−1.2, 0.6)	0.565
CQ6-9-1	1.9 (1.5, 2.4)	0.000	0.0 (−0.8, 0.9)	0.926	−0.3 (−1.2, 0.6)	0.476
CQ6-9-2	0.3 (0.1, 0.5)	0.001	0.2 (−0.4, 0.9)	0.445	−0.2 (−0.9, 0.5)	0.565
CQ6-10-1	1.7 (0.1, 3.3)	0.041	0.4 (−2.9, 3.7)	0.800	2.1 (−1.3, 5.5)	0.223
CQ6-10-2	1.8 (0.5, 3.1)	0.006	1.7 (−1.6, 5.0)	0.297	0.7 (−2.9, 4.3)	0.684
CQ7-1	1.3 (−0.4, 2.9)	0.135	1.3 (−2.0, 4.5)	0.438	0.3 (−2.6, 3.2)	0.829
CQ7-2	0.1 (−0.1, 0.4)	0.389	−0.3 (−0.8, 0.3)	0.300	0.7 (0.0, 1.4)	0.047
CQ7-3	0.3 (0.2, 0.5)	0.000	−0.4 (−0.7, −0.1)	0.022	−0.3 (−0.6, 0.1)	0.164
CQ10-2	0.5 (−0.5, 1.5)	0.294	0.4 (−1.6, 2.4)	0.712	1.2 (−1.8, 4.2)	0.413
CQ11-1	0.6 (0.2, 0.9)	0.001	−0.7 (−1.5, 0.1)	0.070	1.1 (0.3, 1.8)	0.010
CQ11-2	0.2 (0.1, 0.4)	0.001	−0.2 (−0.6, 0.3)	0.469	0.2 (−0.3, 0.6)	0.462
CQ11-3	1.5 (1.2, 1.9)	0.000	−0.4 (−1.5, 0.7)	0.506	0.0 (−1.2, 1.3)	0.951
CQ11-7	1.0 (0.7, 1.2)	0.000	−0.1 (−0.6, 0.3)	0.491	−0.1 (−0.8, 0.6)	0.768
CQ12-2	0.0 (−0.5, 0.5)	0.963	0.7 (−0.5, 1.9)	0.259	0.0 (−1.4, 1.4)	0.965
CQ12-3	−0.6 (−1.4, 0.1)	0.107	−1.0 (−2.7, 0.7)	0.223	0.3 (−1.9, 2.5)	0.796
CQ15-3	−1.7 (−2.5, −0.8)	0.000	1.5 (−0.5, 3.6)	0.134	0.8 (−1.0, 2.5)	0.399
CQ15-5	−1.1 (−2.1, −0.1)	0.030	−1.5 (−4.7, 1.7)	0.356	−0.2 (−4.0, 3.7)	0.932
CQ17-1	2.9 (2.2, 3.5)	0.000	−0.1 (−1.7, 1.6)	0.940	0.3 (−2.0, 2.7)	0.770
CQ21-3	−1.4 (−2.3, −0.5)	0.004	−1.0 (−2.6, 0.5)	0.190	2.8 (1.2, 4.4)	0.001
CQ22-1	−0.4 (−0.7, −0.1)	0.005	0.8 (0.2, 1.3)	0.009	0.2 (−0.3, 0.8)	0.401

Pre-intervention trends and slope changes were reported in years, with 95% CIs

CQ, Clinical Question; CI, Confidence Interval; J-SSCG, Japanese Clinical Practice Guidelines for Management of Sepsis and Septic Shock

### Temporal trends in clinical outcomes: ITS analysis

Adjusted ITS analyses of clinical outcomes revealed that the publication of the J-SSCG 2020 was not associated with a significant change in in-hospital mortality, whereas modest downward slope trends were observed in length of stay and total hospitalization costs (Supplementary material 1 :Tables S6 and Fig. S6).

### Hospital-Level variation in guideline adherence

Hospital-level adherence variation was assessed using ICCs (Table 4). In the post-guideline period, adjusted ICCs exceeded 10% for 22 of the 26 CQs, indicating substantial between-hospital variation. Only four CQs—CQ11–1 (furosemide for AKI), CQ12–2 (enteral nutrition for septic shock), CQ12–3 (early enteral nutrition), and CQ22–1 (antiulcer)—had ICCs < 10%. The highest ICCs were observed for CQ21–3 (ICU admission, 80.4–84.5%), CQ4–9 (procalcitonin, 47.0–53.1%), and CQ6–10–2 (vasopressin, 50.8–51.8%).

**Table 4:** Intraclass correlation coefficients for hospital-level variation in adherence to clinical questions before and after J-SSCG 2020 publication

CQ	Unadjusted ICCs			Adjusted ICCs		
	Before J-SSCG2020	After J-SSCG2020	ICC Change	Before J-SSCG2020	After J-SSCG2020	ICC Change
CQ2-1	19.3	20.1	0.8	20.0	20.6	0.6
CQ4-9	53.6	47.6	−6.1	53.1	47.0	−6.0
CQ5-1	33.0	35.1	2.1	32.0	33.8	1.8
CQ6-1	19.5	22.6	3.1	19.7	22.4	2.6
CQ6-4	23.6	24.4	0.8	23.5	24.2	0.6
CQ6-7	16.2	17.9	1.7	14.5	16.5	1.9
CQ6-8	15.1	19.0	3.9	17.6	23.2	5.6
CQ6-9-1	43.6	51.2	7.6	42.0	50.2	8.2
CQ6-9-2	17.8	19.3	1.5	16.4	18.9	2.5
CQ6-10-1	44.1	41.8	−2.3	43.7	41.7	−2.0
CQ6-10-2	51.7	52.2	0.5	50.8	51.8	1.0
CQ7-1	26.9	25.9	−1.0	25.7	25.4	−0.3
CQ7-2*	–	–	–	–	–	–
CQ7-3	20.5	20.6	0.1	18.9	18.4	−0.5
CQ10-2	23.6	22.9	−0.6	22.3	20.9	−1.4
CQ11-1	5.0	5.0	0.0	4.8	4.3	−0.5
CQ11-2	30.9	33.3	2.5	27.7	29.5	1.8
CQ11-3	21.0	27.0	6.0	24.3	30.1	5.8
CQ11-7	34.4	38.7	4.3	34.7	38.3	3.6
CQ12-2	5.3	5.7	0.3	6.0	6.0	0.0
CQ12-3	5.1	5.4	0.3	5.9	6.1	0.2
CQ15-3	36.9	37.8	0.9	36.5	37.8	1.3
CQ15-5	23.1	26.8	3.7	23.5	26.9	3.3
CQ17-1	18.7	17.6	−1.1	19.7	18.7	−1.0
CQ21-3	83.8	79.1	−4.7	84.5	80.4	−4.2
CQ22-1	9.9	10.2	0.3	8.1	8.3	0.2

ICCs were estimated using multilevel logistic regression models with random intercepts for hospitals. Adjusted ICCs accounted for age, sex, hospital-acquired sepsis, surgery with general anesthesia, infection type, and total SOFA score

*The low occurrence of the outcome caused convergence issues in the multilevel regression models, making ICC calculation unfeasible

ICC, Intraclass Correlation Coefficient; SOFA, Sequential Organ Failure Assessment; J-SSCG, Japanese Clinical Practice Guidelines for Management of Sepsis and Septic Shock

Comparisons of pre- and post-guideline ICCs showed increases in 15 CQs, decreases in 6, and no change in 5. The magnitude of these changes was generally small, with all ICC shifts < 10%, indicating limited change in hospital-level variation over time.

## Discussion

This nationwide study assessed the real-world implementation of the J-SSCG 2020 across 26 clinical recommendations using a large, multicenter inpatient database. Our findings yield three key insights. First, adherence to guideline-recommended care varied substantially by CQ, highlighting a persistent evidence–practice gap in sepsis management. Second, despite the publication of the J-SSCG 2020, changes in adherence rates were minimal—typically less than 3% points—and no abrupt or substantial changes were identified in the ITS analysis, suggesting limited influence of the guideline on clinical practice. Third, hospital-level variation in adherence remained considerable across most recommendations, with adjusted ICCs exceeding 10% for 22 of the 26 CQs. These between-hospital differences did not diminish after the guideline’s publication, indicating that the guideline alone was insufficient to promote nationwide standardization of sepsis care.

This study revealed marked variation in adherence across individual J-SSCG 2020 recommendations. Consistent with previous research [12, 13], adherence was high for recommendations already integrated into clinical practice, such as obtaining blood cultures (CQ2-1), measuring lactate (CQ6-4), administering noradrenaline (CQ6-9), and using antiulcer prophylaxis (CQ22-1). In contrast, adherence was low for recommendations requiring a shift in practice or supported by low-certainty evidence, including procalcitonin (CQ4–9), echocardiography (CQ6–1), fludrocortisone (CQ7–2), antithrombin (CQ15–3), and ICU admission (CQ21-3). Unlike earlier studies limited to 39 or 59 hospitals [12, 13], our investigation evaluated 26 CQs across 791 hospitals, offering a nationally representative and granular overview of real-world adherence. By utilizing a routinely collected administrative database, our approach also enables long-term tracking of practice trends in response to future guideline revisions.

The 45-month study period allowed for a rigorous evaluation of the temporal effects of the J-SSCG 2020—an approach not previously undertaken in Japan. Despite its publication, most CQs exhibited changes of less than 3% points, and ITS analysis revealed no abrupt or substantial shifts in clinical practice. While some CQs demonstrated statistically significant level or slope changes, the observed effects were small, generally less than 1–2% annually. These findings align with previous reports indicating that guideline dissemination alone often fails to trigger rapid behavior change among clinicians [7, 8].

Importantly, non-adherence in our study should not be interpreted uniformly as poor-quality care, particularly in the context of weak recommendations. According to the GRADE framework, weak recommendations (e.g., GRADE 2 or expert consensus), which constituted 24 of the 26 assessed CQs, were intended to allow for clinical discretion depending on individual patient contexts. In this context, non-adherence should not be interpreted as a failure or indicator of poor-quality care. Rather, partial implementation might reflect appropriate individualization of care in line with the flexibility intended by weak recommendations.

Several factors may explain limited effect of guideline publication in this study. First, as summarized in Supplementary material 1: Tables S4,14 of the 26 assessable CQs were consistent with those in the J-SSCG 2016, likely contributing to adherence plateaus and limiting further improvement. However, even among the 12 newly introduced CQs, adherence gains were negligible. Second, many recommendations were weak (e.g., GRADE 2 C/2D or expert consensus), which were intended to support clinical decision-making. In this context, limited adherence might reflect appropriate discretion rather than poor-quality care. Third, structural or resource limitations (e.g., access to echocardiography, ICU beds, or staffing for early rehabilitation) may have hindered the feasibility of implementing certain recommendations. Importantly, a nationwide survey by the J-SSCG 2020 committee reported that 72% of respondents were aware of the guideline, 81% used it in sepsis care, and 95% found it useful [24], suggesting that awareness does not necessarily translate into clinical adoption.

Hospital-level variation in adherence was substantial across most CQs, with adjusted ICCs exceeding 10% for 22 of the 26 evaluated CQs. This indicates that hospital-level factors significantly influenced the delivery of guideline-recommended care, independent of individual patient characteristics. Previous studies in various healthcare contexts have similarly reported high hospital-level variation using ICCs. For instance, a study of heart failure care across 189 hospitals in China found hospital-level factors explained 4.8–21.6% of the adherence variance [25]. In Kenya, pediatric care adherence showed ICCs ranging from 9 to 43% [26]. Such variation may stem from differences in institutional culture, resources, and interpretations of weak or conditional recommendations. Additionally, comparison of ICCs before and after the J-SSCG 2020 publication showed no consistent reduction in between-hospital variability, implying that passive dissemination of the guideline did not reduce institutional disparities. This may be due to passive dissemination without active implementation support, leading to uneven adoption across hospitals. Many recommendations were weak (e.g., GRADE 2 C/2D), which may have allowed for clinical discretion and further variation. Hospitals also differed in baseline practices and readiness to change, further impeding uniform adoption. These findings indicate that guideline publication alone cannot address institutional variability and that system-level interventions warrant further investigation.

This study had several limitations. First, only 26 (22.0%) of the 118 CQs in the J-SSCG 2020 were assessable with the available data, limiting generalizability. Second, although the DPC administrative database has shown good validity in previous studies, it was not designed to assess adherence to specific guideline recommendations. The accuracy of our adherence definitions has not been externally validated. Furthermore, adherence was evaluated based on recorded interventions rather than clinical appropriateness, which may vary by patient context. Third, the SSCG 2016 was replaced by the SSCG 2021 on October 2, 2021 [27], which may have affected clinical practice during the final months of the study. However, according to a nationwide survey by the J-SSCG 2020 committee, a higher proportion of Japanese clinicians reported referencing to the J-SSCG 2020 (88.4%) rather than to the SSCG 2021 (80.8%) during their sepsis care, supporting the relevance of focusing on J-SSCG adherence in our analysis [24]. Lastly, the coronavirus disease 2019 pandemic overlapped with the publication and implementation of the J-SSCG 2020 and might have influenced clinical priorities during this period. This might have interfered with the adoption of recommendations and affected adherence trends, independent of the guidelines’ content.

## Conclusions

This nationwide study demonstrated considerable variation in adherence to the J-SSCG 2020 across clinical recommendations, limited change in clinical practice following guideline publication, and persistent hospital-level differences. These findings emphasize the challenges of translating guidelines into practice. Given that most assessed recommendations were weak and designed to allow for clinical discretion, partial adherence might not necessarily indicate poor-quality care. Future research should focus on identifying barriers to standardized sepsis care and addressing institutional factors contributing to variation.

## Supplementary Information

Supplementary Material 1.

## Data Availability

The data used in the manuscript will not be made available because the datasets analysed are not publicly available, owing to contracts with the hospitals that provided the data.

## Abbreviations

J-SSCG: Japanese Clinical Practice Guidelines for Management of Sepsis and Septic Shock
SSCG: Surviving Sepsis Campaign
CQ: Clinical Question
ICU: Intensive care unit
HDU: High-dependency unit
DPC: Diagnosis Procedure Combination
SOFA: Sequential Organ Failure Assessment
ICD-10: International Classification of Diseases, 10th Revision
ITS: Interrupted time series
ICC: Intraclass correlation coefficient
PMX-DHP: Polymyxin B-immobilized fiber column direct hemoperfusion
AKI: Acute kidney injury
DIC: Disseminated intravascular coagulation
