# Supplementary material for: Impact of the Japanese clinical practice guidelines for management of sepsis and septic shock (J-SSCG) 2020 on real-world adherence and interhospital variation: a nationwide inpatient database study
Source: Crit Care. 2025 Jun 3;29:225. doi: 10.1186/s13054-025-05482-9 (PMC12135573; doi:10.1186/s13054-025-05482-9)
Supplement: Supplementary file 1 — Supplementary Material 1. [file 13054_2025_5482_MOESM1_ESM.docx]

**Supplemental materials**

**Impact of the Japanese Clinical Practice Guidelines for Management of Sepsis and Septic Shock (J-SSCG) 2020 on real-world adherence and interhospital variation: a nationwide inpatient database study**

Hiroyuki Ohbe^1,2^**^*^**, Kazuma Yamakawa^3^, Daisuke Kudo^1,4^, Shotaro Aso^5^, Hiroki Matsui^2^, Kiyohide Fushimi^6^, Hideo Yasunaga^2^, Tomoaki Yatabe^7^, Moritoki Egi^8^, Hiroshi Ogura^9^, Osamu Nishida^10^, Shigeki Kushimoto^4,11^

^1^Department of Emergency and Critical Care Medicine, Tohoku University Hospital, 1-1 Seiryo-machi, Aoba-ku, Sendai 980-8574, Japan

^2^Department of Clinical Epidemiology and Health Economics, School of Public Health, The University of Tokyo, 7-3-1 Hongo, Bunkyo-ku, Tokyo 113-0033, Japan

^3^Department of Emergency and Critical Care Medicine, Osaka Medical and Pharmaceutical University, 2-7 Daigaku-machi, Takatsuki, Osaka 569-8686, Japan.

^4^Division of Emergency and Critical Care Medicine, Tohoku University Graduate School of Medicine, 2-1 Seiryo-machi, Aoba-ku, Sendai, Miyagi, 980-8575, Japan

^5^Department of Real-world Evidence, Graduate School of Medicine, The University of Tokyo, 7-3-1, Hongo, Bunkyo-ku, Tokyo, 113-8655, Japan.

^6^Department of Health Policy and Informatics, Institute of Science Tokyo Graduate School, 2-12-1 Ookayama, Meguro-ku, Tokyo 152-8550, Japan

^7^Emergency Department, Nishichita General Hospital, 3-1-1 Nakanoike, Tokai-shi, Aichi 477-8522, Japan.

^8^Department of Anesthesia and Intensive Care, Kyoto University Hospital, 54 Kawahara-cho, Shogoin, Sakyo-ku, Kyoto, 606-8507, Japan.

^9^Department of Clinical Laboratory, Osaka General Medical Center, 3-1-56 Bandaicho, Sumiyoshi-ku, Osaka-shi, Osaka 558-8558, Japan

^10^Department of Anesthesiology and Critical Care Medicine, Fujita Health University, 1-98 Dengakugakubo, Kutsukake, Toyoake, Aichi 470-1192, Japan

^11^Executive Advisor for Emergency Medicine and Intensive Care, Kawasaki Saiwai Hospital, 31-27 Omiya-cho, Saiwai-ku, Kawasaki, Kanagawa 212-0014, Japan

**^*^Corresponding author:** Hiroyuki Ohbe

Department of Emergency and Critical Care Medicine, Tohoku University Hospital, 1-1 Seiryo-machi, Aoba-ku, Sendai 980-8574, Japan

E-mail: [hohbey@gmail.com](mailto:hohbey@gmail.com); Tel: +81-2-2717-7489; Fax: +81-2-2717-7492

**Supplemental Table 1.** Definitions of numerators and denominators for assessable clinical questions in the J-SSCG 2020

| Clinical Question | Numerator | Denominator |
| --- | --- | --- |
| CQ2-1: When should a blood culture be taken? | Number of patients who underwent blood culture collection on or before the first day of sepsis treatment | Number of patients with sepsis |
| CQ4-9: Should PCT be used as an indicator for stopping antimicrobial therapy for sepsis? | Number of patients who received three or more procalcitonin tests within 14 days of the first day of sepsis treatment | Number of patients with sepsis who survived ≥5 days from the first day of sepsis treatment |
| CQ5-1: Should intravenous immunoglobulin be administered to adult patients with sepsis? | Number of patients who did not receive intravenous immunoglobulin during hospitalization | Number of patients with sepsis |
| CQ6-1: Should echocardiography be conducted in patients with sepsis? | Number of patients who underwent echocardiography on or before the first day of sepsis treatment | Number of patients with septic shock on the first day of sepsis treatment |
| CQ6-4: Should lactate levels be used as an indicator for initial resuscitation in adult patients with sepsis? | Number of patients who received a lactate test (blood gas analysis) on the first day of sepsis treatment | Number of patients with septic shock on the first day of sepsis treatment |
| CQ6-7: Should albumin solution be used for initial resuscitation in adult patients with sepsis? | Number of patients who did not receive albumin solution on the first day of sepsis treatment | Number of patients with septic shock on the first day of sepsis treatment |
| CQ6-8: Should artificial colloids be used for initial resuscitation in adult patients with sepsis? | Number of patients who did not receive artificial colloids on the first day of sepsis treatment | Number of patients with septic shock on the first day of sepsis treatment |
| CQ6-9-1: Should noradrenaline, dopamine, or phenylephrine be used as a first-line vasopressor in adult patients with sepsis? noradrenaline vs. dopamine | Number of patients who received noradrenaline on the first day of sepsis treatment | Number of patients who received either noradrenaline or dopamine on the first day of sepsis treatment |
| CQ6-9-2: Should noradrenaline, dopamine, or phenylephrine be used as a first-line vasopressor in adult patients with sepsis? noradrenaline vs. phenylephrine | Number of patients who received noradrenaline on the first day of sepsis treatment | Number of patients who received either noradrenaline or phenylephrine on the first day of sepsis treatment |
| CQ6-10-1: Should adrenaline be used as a second-line vasopressor in adult patients with sepsis? | Number of patients who did not receive adrenaline on the first day of sepsis treatment | Number of patients who received second-line vasopressor (either adrenaline or vasopressin) on the first day of sepsis treatment |
| CQ6-10-2: Should vasopressin be used as a second-line vasopressor in adult patients with sepsis? | Number of patients who received vasopressin on the first day of sepsis treatment | Number of patients who received second-line vasopressor (either adrenaline or vasopressin) on the first day of sepsis treatment |
| CQ7-1: Should low-dose corticosteroids (hydrocortisone) be administered to adult patients with septic shock who do not respond to initial fluid resuscitation and vasopressors? | Number of patients who received low-dose corticosteroids on the first day of sepsis treatment | Number of patients who received second-line vasopressor (either adrenaline or vasopressin) on the first day of sepsis treatment |
| CQ7-2: Should hydrocortisone and fludrocortisone be administered to patients with septic shock who do not respond to initial fluid resuscitation and vasopressors? | Number of patients who received fludrocortisone on the first day of sepsis treatment | Number of patients who received low-dose corticosteroids (hydrocortisone) and second-line vasopressor (either adrenaline or vasopressin) on the first day of sepsis treatment |
| CQ7-3: Should corticosteroids (hydrocortisone) be administered to patients with sepsis without shock? | Number of patients who received low-dose corticosteroids on the first day of sepsis treatment | Number of patients with sepsis but not septic shock on the first day of sepsis treatment |
| CQ10-2: Should propofol or dexmedetomidine be prioritized over benzodiazepines as sedatives for adult patients with sepsis on mechanical ventilation? | Number of patients who received propofol or dexmedetomidine within 14 days of the first day of sepsis treatment | Number of patients who received invasive mechanical ventilation and sedatives within 14 days of the first day of sepsis treatment |
| CQ11-1: Should furosemide be used to prevent or treat septic AKI? | Number of patients who did not receive furosemide on the first day of sepsis treatment | Number of patients whose renal SOFA score ≥ 1 on the first day of sepsis treatment |
| CQ11-2: Should atrial natriuretic peptide (ANP) be used to prevent or treat septic AKI? | Number of patients who did not receive ANP on the first day of sepsis treatment | Number of patients whose renal SOFA score ≥ 1 on the first day of sepsis treatment |
| CQ11-3: Should dopamine be used to prevent or treat septic AKI? | Number of patients who did not receive dopamine on the first day of sepsis treatment | Number of patients whose renal SOFA score ≥ 1 on the first day of sepsis treatment |
| CQ11-7: Should PMX-DHP be used for patients with septic shock? | Number of patients who did not receive PMX-DHP on the first day of sepsis treatment | Number of patients with septic shock on the first day of sepsis treatment |
| CQ12-2: Should hemodynamically unstable septic shock patients receive enteral nutrition? | Number of patients who did not receive enteral nutrition on the first day of sepsis treatment | Number of patients with septic shock on the first day of sepsis treatment |
| CQ12-3: When should enteral nutrition be initiated in septic patients? | Number of patients who received enteral nutrition within 2 days of the first day of sepsis treatment | Number of patients with sepsis |
| CQ15-3: Should antithrombin replacement therapy be administered in sepsis-associated DIC? | Number of patients who received antithrombin during hospitalization | Number of patients diagnosed with DIC |
| CQ15-5: Should recombinant thrombomodulin be administered to patients with sepsis-associated DIC? | Number of patients who received recombinant thrombomodulin during hospitalization | Number of patients diagnosed with DIC |
| CQ17-1: Should early rehabilitation be implemented to prevent PICS? | Number of patients who received rehabilitation within 7 days of the first day of sepsis treatment | Number of patients with sepsis |
| CQ21-3: Where should sepsis which does not respond to initial fluid resuscitation be managed? | Number of patients admitted to ICU on the first day of sepsis treatment | Number of patients with septic shock on the first day of sepsis treatment |
| CQ22-1: Should antiulcer drugs be administered to septic patients to prevent gastrointestinal bleeding? | Number of patients who received antiulcer drugs (H2 blocker or PPI) during hospitalization | Number of patients with sepsis |

This table summarizes how each of the 26 assessable clinical questions (CQs) in the J-SSCG 2020 was operationalized using data from the Diagnosis Procedure Combination database. In this study, septic shock was defined as the use of any vasopressor—noradrenaline, adrenaline, dopamine, vasopressin, or phenylephrine—on the first day of sepsis treatment, due to the unavailability of mean arterial pressure and lactate data in the DPC database. CQ, Clinical Question; J-SSCG, Japanese Clinical Practice Guidelines for Management of Sepsis and Septic Shock; DPC, Diagnosis Procedure Combination.

**Supplemental Table 2.** ICD-10 codes for types of infection.

| ICD-10 codes |
| --- |
| **Lung** |
| A15 A16 A31 A37 B371 B440 B441 B450 J01 J02 J03 J04 J05 J06 J13 J14 J15 J16 J17 J18 J20 J21 J22 J440 J441 J47 J69 J85 J86 |
| **Abdomen** |
| A020 A04 A05 A08 A09 A183 K35 K36 K37 K57 K61 K630 K631 K65 K750 K751 K800 K801 K803 K804 K808 K810 K830 K918 |
| **Urinary tract** |
| N10 N11 N12 N151 N159 N160 N30 N34 N390 N41 N74 N75 T835 T836 |
| **Central nervous system** |
| A17 A39 G00 G01 G02 G03 G04 G05 G06 G07 G08 G09 |
| **Skin and soft tissues** |
| A33 A34 A35 A46 A48 B35 B36 L03 L04 L08 L726 L88 |
| **Cardiovascular system** |
| I30 I33 I80 T826 T827 |
| **Others** |
| None of above |

ICD-10 = International Classification of Diseases, 10th Revision

**Supplemental Table 3.** Hospital characteristics (fiscal year 2018–2021).

|  | Fiscal | Fiscal | Fiscal | Fiscal |  |
| --- | --- | --- | --- | --- | --- |
|  | Year | year | year | year | SMD |
|  | 2018 | 2019 | 2020 | 2021 | 2018 vs. |
| Hospital characteristics | N=791 | N=791 | N=791 | N=791 | 2021 |
| Teaching hospital, n (%) | 657 (83.1) | 662 (83.7) | 664 (83.9) | 664 (83.9) | 1.0 |
| Academic hospital, n (%) | 72 (9.1) | 70 (8.8) | 70 (8.8) | 71 (9.0) | -2.7 |
| Tertiary emergency hospital, n (%) | 205 (25.9) | 214 (27.1) | 215 (27.2) | 219 (27.7) | -0.3 |
| Number of acute-care beds, beds | 360 (248–522) | 351 (243–510) | 342 (225–500) | 342 (231–500) | -7.8 |
| Number of ICU beds, beds | 0 (0–10) | 0 (0–10) | 0 (0–9) | 0 (0–10) | -1.5 |
| Annual hospital sepsis case volume | 52 (28–89) | 56 (27–97) | 53 (28–94) | 44 (20–75)* | – |

Continuous variables were presented as means and standard deviations or medians and interquartile ranges, and categorical variables were presented as numbers and percentages, as appropriate.

*Fiscal year 2021 was 9 months.

SMD, standardized mean difference; IQR, interquartile range; ICU, intensive care unit; HDU, high-dependency care unit.

**Supplemental Table 4.** Comparison of J-SSCG 2020 clinical questions with J-SSCG 2016

| CQs in J-SSCG 2020 | Exists in J-SSCG 2016 | Change/New in J-SSCG 2020 |
| --- | --- | --- |
| CQ2-1 | Yes | No major change |
| CQ4-9 | No | New |
| CQ5-1 | Yes | Strengthened against |
| CQ6-1 | Yes | No major change |
| CQ6-4 | Yes | No major change |
| CQ6-7 | Yes | No major change |
| CQ6-8 | Yes | No major change |
| CQ6-9-1 | Yes | No major change |
| CQ6-9-2 | Yes | No major change |
| CQ6-10-1 | No | New |
| CQ6-10-2 | No | New |
| CQ7-1 | Yes | No major change |
| CQ7-2 | No | New |
| CQ7-3 | Yes | No major change |
| CQ10-2 | No | New |
| CQ11-1 | Yes | No major change |
| CQ11-2 | No | New |
| CQ11-3 | Yes | No major change |
| CQ11-7 | No | New |
| CQ12-2 | No | New |
| CQ12-3 | No | New |
| CQ15-3 | Yes | No major change |
| CQ15-5 | Yes | No major change |
| CQ17-1 | No | New |
| CQ21-3 | No | New |
| CQ22-1 | No | New |

“Exist in J-SSCG 2016” indicates whether a similar clinical question was included in the 2016 edition of the guidelines.

“Change/New in 2020” refers to whether the recommendation in J-SSCG 2020 differed in content or direction compared with J-SSCG 2016 (e.g., newly added CQ, reversed recommendation, change in GRADE level, or modified scope).

CQ = Clinical Question; J-SSCG = Japanese Clinical Practice Guidelines for Management of Sepsis and Septic Shock.

**Supplemental Table 5.** Sensitivity interrupted time series analysis redefining post-guideline period to March 2021–December 2021

|  | Pre-trend | P | Level-change | P | Slope change | P |
| --- | --- | --- | --- | --- | --- | --- |
| CQ | (95% CI) | value | (95% CI) | value | (95% CI) | value |
| CQ2-1 | 0.0 (-0.4, 0.4) | 0.954 | 0.8 (-0.5, 2.2) | 0.217 | 0.6 (-1.6, 2.8) | 0.579 |
| CQ4-9 | -0.3 (-0.6, 0.1) | 0.138 | 0.3 (-0.4, 1.1) | 0.367 | 1.7 (0.3, 3.0) | 0.017 |
| CQ5-1 | 1.6 (1.3, 1.9) | 0.000 | -0.1 (-0.7, 0.5) | 0.731 | -0.7 (-1.8, 0.5) | 0.255 |
| CQ6-1 | -0.2 (-0.6, 0.3) | 0.490 | -1.1 (-2.9, 0.7) | 0.235 | 2.5 (0.5, 4.5) | 0.005 |
| CQ6-4 | 0.1 (-0.4, 0.5) | 0.785 | 0.8 (-1.3, 2.9) | 0.461 | -1.1 (-4.8, 2.6) | 0.557 |
| CQ6-7 | 0.5 (0.1, 0.9) | 0.021 | -0.1 (-1.1, 0.9) | 0.800 | 1.3 (0.1, 2.6) | 0.034 |
| CQ6-8 | 0.6 (0.4, 0.9) | 0.000 | -0.8 (-1.5, -0.2) | 0.016 | 1.4 (0.1, 2.7) | 0.038 |
| CQ6-9-1 | 1.9 (1.5, 2.3) | 0.000 | 0.2 (-0.5, 0.8) | 0.597 | -0.7 (-1.7, 0.3) | 0.161 |
| CQ6-9-2 | 0.4 (0.2, 0.6) | 0.000 | -0.4 (-1.2, 0.5) | 0.387 | 0.5 (-0.8, 1.8) | 0.457 |
| CQ6-10-1 | 2.0 (0.8, 3.1) | 0.001 | 0.5 (-4.5, 5.6) | 0.829 | 2.0 (-7.0, 11.0) | 0.653 |
| CQ6-10-2 | 2.6 (1.4, 3.7) | 0.000 | -0.8 (-4.9, 3.2) | 0.680 | 2.7 (-4.1, 9.4) | 0.431 |
| CQ7-1 | 1.6 (0.5, 2.8) | 0.007 | 0.6 (-3.6, 4.8) | 0.775 | 0.1 (-6.6, 6.9) | 0.969 |
| CQ7-2 | 0.1 (-0.1, 0.3) | 0.563 | 0.2 (-0.3, 0.7) | 0.421 | 0.6 (-0.1, 1.3) | 0.095 |
| CQ7-3 | 0.2 (0.1, 0.3) | 0.003 | -0.1 (-0.6, 0.3) | 0.535 | -0.4 (-1.2, 0.4) | 0.357 |
| CQ10-2 | 0.7 (0.0, 1.4) | 0.052 | 0.3 (-1.2, 1.8) | 0.683 | 1.5 (-2.9, 6.0) | 0.488 |
| CQ11-1 | 0.4 (0.1, 0.7) | 0.004 | -0.2 (-1.1, 0.8) | 0.719 | 1.8 (0.3, 3.2) | 0.020 |
| CQ11-2 | 0.2 (0.1, 0.3) | 0.007 | 0.2 (-0.2, 0.5) | 0.328 | 0.1 (-0.4, 0.5) | 0.793 |
| CQ11-3 | 1.4 (1.0, 1.8) | 0.000 | 0.3 (-1.0, 1.6) | 0.663 | -0.5 (-2.6, 1.6) | 0.648 |
| CQ11-7 | 0.9 (0.7, 1.1) | 0.000 | -0.1 (-0.6, 0.4) | 0.765 | -0.1 (-1.3, 1.0) | 0.814 |
| CQ12-2 | 0.2 (-0.2, 0.6) | 0.302 | 0.2 (-1.5, 1.9) | 0.816 | -0.2 (-3.4, 2.9) | 0.874 |
| CQ12-3 | -1.0 (-1.6, -0.4) | 0.001 | 0.6 (-1.1, 2.3) | 0.506 | -0.9 (-4.3, 2.6) | 0.623 |
| CQ15-3 | -1.3 (-1.9, -0.6) | 0.001 | 0.5 (-1.2, 2.1) | 0.571 | 1.6 (0.0, 3.3) | 0.046 |
| CQ15-5 | -1.6 (-2.6, -0.7) | 0.001 | 1.1 (-2.2, 4.3) | 0.512 | -3.0 (-8.8, 2.8) | 0.300 |
| CQ17-1 | 2.9 (2.3, 3.4) | 0.000 | -0.2 (-1.8, 1.4) | 0.795 | 1.1 (-3.0, 5.3) | 0.582 |
| CQ21-3 | -1.6 (-2.2, -1.0) | 0.000 | 1.1 (-0.5, 2.7) | 0.184 | 2.1 (-0.8, 4.9) | 0.154 |
| CQ22-1 | -0.2 (-0.4, 0.0) | 0.030 | 0.8 (0.0, 1.6) | 0.055 | -0.6 (-2.0, 0.9) | 0.449 |

Pre-intervention trends and slope changes were reported in years, with 95% CIs.

CQ, Clinical Question; CI, Confidence Interval; J-SSCG, Japanese Clinical Practice Guidelines for Management of Sepsis and Septic Shock

**Supplemental Table 6.** Interrupted time series analysis of temporal trends in clinical outcomes before and after J-SSCG 2020 publication

|  | Pre-trend | P | Level-change | P | Slope change | P |
| --- | --- | --- | --- | --- | --- | --- |
| Outcomes | (95% CI) | value | (95% CI) | value | (95% CI) | value |
| Mortality | 0.2 (-1.0, 1.3) | 0.789 | 1.6 (-0.9, 4.1) | 0.205 | -1.1 (-4.2, 2.1) | 0.506 |
| LOS | -1.7 (-2.2, -1.1) | 0.000 | 0.0 (-0.8, 0.9) | 0.930 | -1.5 (-2.8, -0.1) | 0.037 |
| Costs | -0.04 (-0.06, -0.02) | 0.002 | 0.07 (0.00, 0.14) | 0.053 | -0.14 (-0.24, -0.03) | 0.014 |

Pre-intervention trends and slope changes were reported in years, with 95% CIs.

CI, Confidence Interval; LOS, Length of Stay; J-SSCG, Japanese Clinical Practice Guidelines for Management of Sepsis and Septic Shock

**Supplemental Figure 1.** Temporal trends in clinical outcomes before and after J-SSCG 2020 publication

The vertical dashed line indicates the publication date of the guideline (October 2020). Data points represent monthly adjusted outcomes using multilevel regression models: in-hospital mortality (top), length of hospital stay (middle), and total hospitalization costs (bottom). Solid red lines represent fitted segmented regression models. The red dashed line depicts the projected outcome trend after October 2020, assuming that the pre-guideline trend had continued unchanged.
